# Nasopharyngeal Lymphoma: A 22-Year Review of 35 Cases

**DOI:** 10.3390/jcm8101604

**Published:** 2019-10-03

**Authors:** Chien-Yu Hsueh, Ching-Fen Yang, Jyh-Pyng Gau, Edward C. Kuan, Ching-Yin Ho, Tzeon-Jye Chiou, Liang-Tsai Hsiao, Ting-An Lin, Ming-Ying Lan

**Affiliations:** 1 Department of Otorhinolaryngology—Head and Neck Surgery, Taipei Veterans General Hospital, Taipei 11217, Taiwan; 2 School of Medicine, National Yang-Ming University, Taipei 11221, Taiwan; cfyang@vghtpe.gov.tw (C.-F.Y.); jpgau@vghtpe.gov.tw (J.-P.G.); tigertpe@gmail.com (C.-Y.H.); tjchiou@vghtpe.gov.tw (T.-J.C.); lthsiao@vghtpe.gov.tw (L.-T.H.); tinganlin1008@gmail.com (T.-A.L.); 3 Department of Pathology and Laboratory Medicine, Taipei Veterans General Hospital, Taipei 11217, Taiwan; 4 Division of Hematology and Oncology, Department of Medicine, Taipei Veterans General Hospital, Taipei 11217, Taiwan; 5 Department of Otolaryngology—Head and Neck Surgery, University of California, Irvine, Orange, CA 92868, USA; eckuan@hs.uci.edu; 6 Department of Otolaryngology, Cheng Hsin Hospital, Taipei 11220, Taiwan; 7 Division of Transfusion Medicine, Department of Medicine, Taipei Veterans General Hospital, Taipei 11217, Taiwan

**Keywords:** nasopharynx, lymphoma, EBER, DLBCL, NKTCL

## Abstract

Nasopharyngeal (NP) lymphoma is a rare primary malignancy of the head and neck and represents a minority of malignancies originating from the nasopharynx. For this reason, there are limited data regarding epidemiologic and treatment outcomes. This is a retrospective review of patients diagnosed with NP lymphoma from 1995 to 2017 at a tertiary medical center. The patients’ demographic data, clinical presentations, treatment modalities, Epstein–Barr virus (EBV)-encoded small RNA (EBER) staining, and outcomes were investigated. We considered a total of 35 patients, including 20 males and 15 females, diagnosed with NP lymphoma. The age ranged from 17 to 88 years (mean = 59.6). The common presentations were nasal obstruction, epistaxis, and neck mass. In our study, the most common pathological diagnosis of NP lymphoma was diffuse large B cell lymphoma (DLBCL) (*n* = 17), followed by NK/T cell lymphoma (NKTCL) (*n* = 9). Other pathologic diagnoses included extranodal marginal zone lymphoma of mucosa-associated lymphoid tissue (MALToma), small lymphocytic lymphoma, mantle cell lymphoma. There were 13 cases showing EBER positivity, including 7 cases of NKTCL, 5 cases of DLBCL, and 1 case of post-transplant lymphoproliferative disorder (PTLD). Most patients received chemotherapy alone, while some patients received both chemotherapy and radiotherapy. Seven patients had local recurrence, and fewer than half of the patients (*n* = 16) were alive at the time of the study (mean follow-up duration: 54.4 months). The five-year overall survival was 50.4%. NP lymphoma is very rare, and the most common pathologic type is DLBCL. EBER positivity is found in both NKTCL and DLBCL. Identifying more effective therapeutic agents is extremely important to improve patients’ survival.

## 1. Introduction

Lymphoma, the most common non-epithelial malignancy of the head and neck region, can be classified into two types, Hodgkin’s lymphoma (HL) and non-Hodgkin’s lymphoma (NHL) [1]. Among them, primary nasopharyngeal (NP) lymphoma is a very rare tumor, with only limited studies found in the literatures [2,3,4,5,6,7,8,9,10]. Waldeyer-Hartz firstly described the lymphoid tissues within the nasopharynx, oropharyngeal wall, base of the tongue, palatine tonsils, and soft palate, known today as Waldeyer’s ring (WR) [7]. It has been reported that less than 10% of NHL cases in Western countries involved the WR (WR-NHL) [11,12,13], while 10–18% of NHL cases were WR-NHL in Asian countries [7]. Among WR-NHL, about 35–37% of cases were at the nasopharyngeal site, and there was no difference of incidence between Western and Asian countries [7,9].

NP lymphoma is a rare extranodal lymphoma with a variety of histopathologic subtypes, and the incidences of different subtypes are quite different among different areas [7,9]. Diffuse large B cell lymphoma (DLBCL) and NK/T cell lymphoma (NKTCL) are the two most common pathologic diagnoses [7,9]. Epstein–Barr virus (EBV) has been known to be related to lymphoma pathogenesis. However, related studies in NP lymphoma are very few. EBV-associated viral proteins were found to play important roles in the genesis of lymphomas through several mechanisms [10,14,15,16,17]. The expression of EBV-encoded small RNAs (EBER) is common in NKTCL, while EBER positivity is still controversial in DLBCL [10,18,19,20,21,22]. There are studies reporting that EBER-positive patients have a poorer prognosis than EBER-negative patients [10,23].

So far, there are relatively few data regarding NP lymphomas, especially in areas with high incidence of nasopharyngeal carcinoma (NPC). The aim of this study was to analyze the clinicopathologic characteristics and survival of NP lymphoma patients at a tertiary medical center in Taiwan. Subgroup analyses was also conducted to further identify the association between EBER positivity and the clinical features of NP lymphomas. This is one of the only two studies investigating EBER expression in NP lymphoma [4].

## 2. Materials and Methods

### 2.1. Patient Population

We retrospectively reviewed the records and data of patients who had undergone NP biopsy demonstrating pathology-proven lymphoma at Taipei Veterans General Hospital in Taipei, Taiwan, between 1 January, 1995 and 31 October, 2017. The Institutional Review Board (IRB) of Taipei Veterans General Hospital approved this study. All procedures performed in studies involving human participants were in accordance with the ethical standards of the institutional and/or national research committee and with the 1964 Helsinki declaration and its later amendments or comparable ethical standards.

The diagnostic criteria for lymphoma were based on the World Health Organization classification [1] using both morphologic and immunohistochemical evaluations. All patients were staged according to the Ann Arbor staging system [24]. Data including sex, age, tumor stage (according to Ann Arbor classification), underlying medical comorbidities (hypertension, diabetes, or coronary artery disease), initial clinical presentation, duration of symptoms, pathology, treatment modalities and outcomes, and survival time, were reviewed. Immunohistochemistry (IHC) and EBERs in situ hybridization (ISH) examinations were used for pathologic diagnoses. The diagnoses of all cases were reviewed and confirmed.

### 2.2. ISH of EBERs

ISH studies were performed on formalin-fixed, paraffin-embedded tissues. Tissue sections were deparaffinized according to established procedures. Antigen retrieval was performed using the Enzyme Pretreatment Kit (AR9551, Leica Biosystems Newcastle Ltd, UK) for 5 min at 37 °C. We used the Bond™ Ready-to-Use ISH EBER Probe (PB0589, Leica Biosystems Newcastle Ltd, UK) as the primary antibody for 90 minutes. Slides were then stained using Bond™ Polymer Refine Detection (DS9800, Leica Biosystems Newcastle Ltd, UK) with the Leica Biosystems BONDMAX autostainer (Leica Biosystems Newcastle Ltd, UK), according to the manufacturer’s instructions. Briefly, an anti-fluorescein antibody was used for 15 minutes, followed by the post-primary IgG linker reagent which localized the mouse antibody for 4 minutes, and the poly-HRP IgG reagent which localized the rabbit antibody for 4 minutes. Staining was then developed with the substrate chromogen DAB (3,3’-diaminobenzidine tetrahydrochloride) for 10 minutes. Finally, the sections were counterstained with modified Mayer’s hematoxylin for 10 minutes.

### 2.3. Statistical Analysis

Quantitative data were summarized as mean ± standard deviation (SD), and categorical variables as percentages. IBM SPSS 20.0 software (IBM Corp, Armonk, NY, USA) was used for statistical analyses. Categorical data were compared using the chi-square test. The Kaplan–Meier method was used for estimating overall survival (OS), defined as the time in months from diagnosis to death or last follow-up due to any cause. Differences in survival were compared using the log-rank test. All tests were two-tailed and conducted considering a 5% significance level.

## 3. Results

### 3.1. Patients

We considered a total of 35 patients, including 20 males and 15 females, diagnosed with NP lymphoma at Taipei Veterans General Hospital between 1995 and 2017. The age at diagnosis ranged from 17 to 88 years (mean 59.6). Ten patients had hypertension, six had diabetes, and four had coronary artery disease. The most common presenting signs and symptoms included nasal obstruction (28.9%, *n* = 10), epistaxis (25.7%, *n* = 9), neck mass (22.9%, *n* = 8), purulent rhinorrhea (17.1%, *n* = 6), headache (14.3%, *n* = 5), and B symptoms (14.3%, *n* = 5) (Table 1). The average time window between initial symptoms to diagnosis ranged from 0.5 to 12 months (mean 2.6 months). The average white blood cell count (WBC) was 12,992 cells/cm^3^ (2400–167,800), and the average lactic dehydrogenase (LDH) level was 337.7 U/L (106–1702).

**Figure 1:**
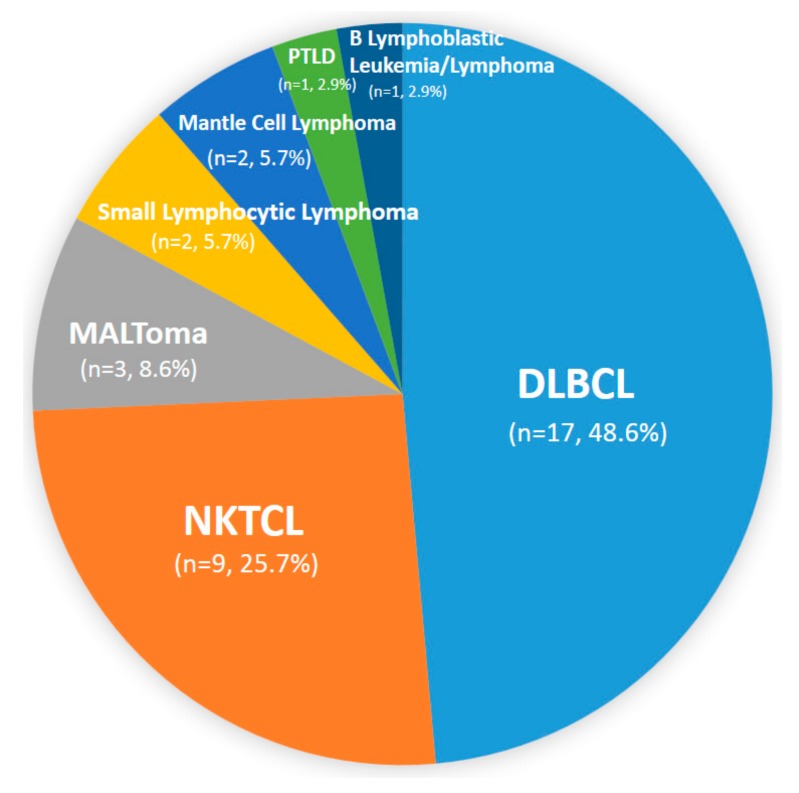
Histological subtypes of nasopharyngeal (NP) lymphoma. Abbreviations: DLBCL: diffuse large B cell lymphoma; NKTCL: NK/T cell lymphoma; MALToma: extranodal marginal zone lymphoma of mucosa-associated lymphoid tissue; PTLD: post-transplant lymphoproliferative disorder.

In our study, the most common pathological diagnosis of nasopharyngeal lymphoma was DLBCL (48.6%, *n* = 17), followed by NKTCL (25.7%, *n* = 9). Other pathologic diagnoses included extranodal marginal zone lymphoma of mucosa-associated lymphoid tissue (MALToma) (8.6%, *n* = 3), small lymphocytic lymphoma (5.7%, *n* = 2), mantle cell lymphoma (5.7%, *n* = 2), B lymphoblastic leukemia/lymphoma (2.9%, *n* = 1), and post-transplant lymphoproliferative disorder (PTLD) (2.9%, *n* = 1) (Figure 1).

**Figure 2:**
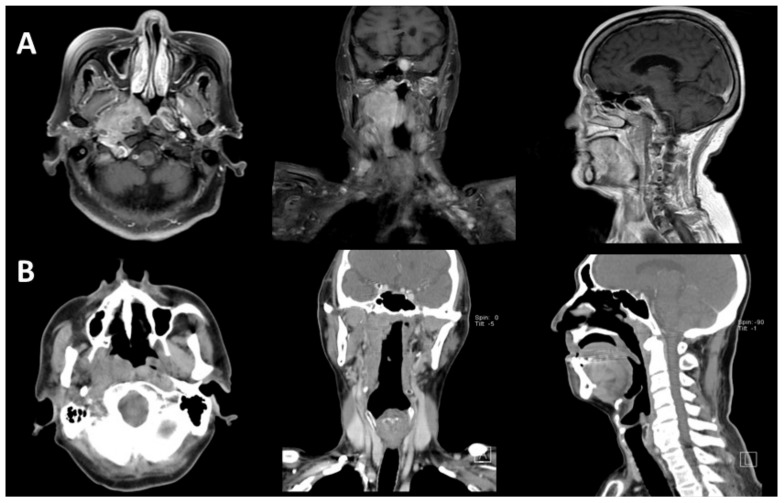
MRI and CT images of NP lymphomas. (**A**) An 85-year-old female diagnosed with NP DLBCL. The MRI revealed that the tumor involved the right NP and right parapharyngeal space. (**B**) A 73-year-old male diagnosed with NP NKTCL. The CT scan revealed that the tumor involved the right NP.

**Figure 3:**
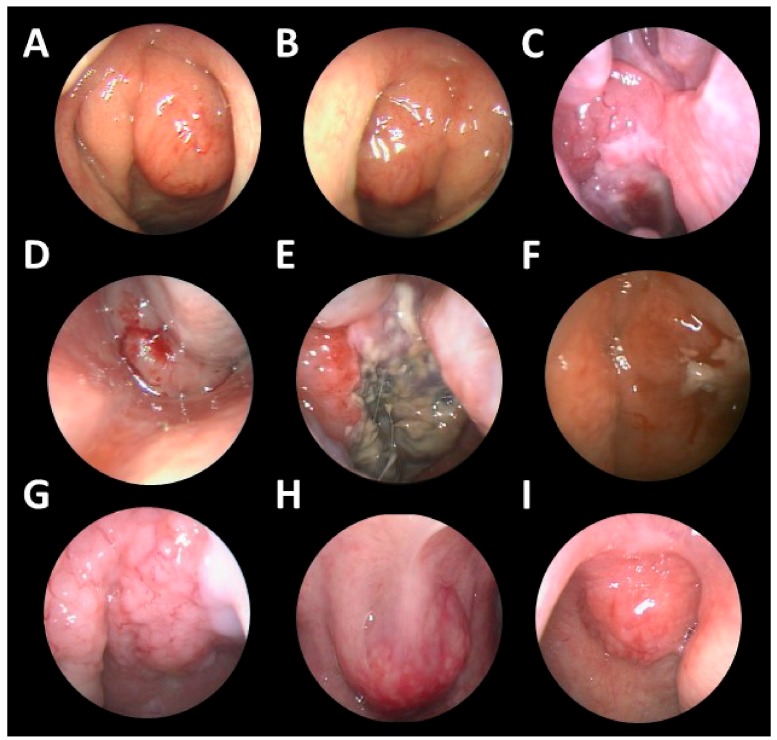
Endoscopic views of various NP lymphomas. Endoscopic examinations showed various NP lymphomas, which were heterogeneous, from smooth bulging masses to hemorrhagic masses, and some tumors showed necrosis over their surface: (**A**,**B**) small lymphocytic lymphoma, (**C**,**D**,**E**) DLBCL, (F) NKTCL, (**G**,**E**) mantle cell lymphoma, (**I**) MALToma.

As for tumor location, most of the tumors (62.9%, *n* = 22) were located in the nasopharynx only (Figure 2 and Figure 3). Some of the tumors (*n* = 3) extended to adjacent sites, including the paranasal sinuses, nasal cavity, oral cavity, and oropharynx. Ten cases (28.6%) had neck lymph nodes involvement or other distant involvements when diagnosed. On the basis of the Ann Arbor staging system, 12 patients (34.3%) had stage I, 15 patients (42.9%) had stage II, and 8 patients (22.9%) had stage IV lymphoma. The characteristics of stage IV cases are listed in Supplementary Table S1. There were no cases with central nervous system (CNS) involvement in our series.

### 3.2. EBERs

**Figure 4:**
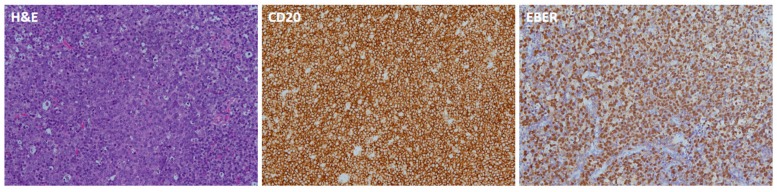
Pathologic images of NP-DLBCL. The hematoxylin and eosin (H&E)-stained slide shows diffuse proliferation of large atypical lymphoid cells. The neoplastic cells are positive for CD20 and EBER.

**Figure 5:**
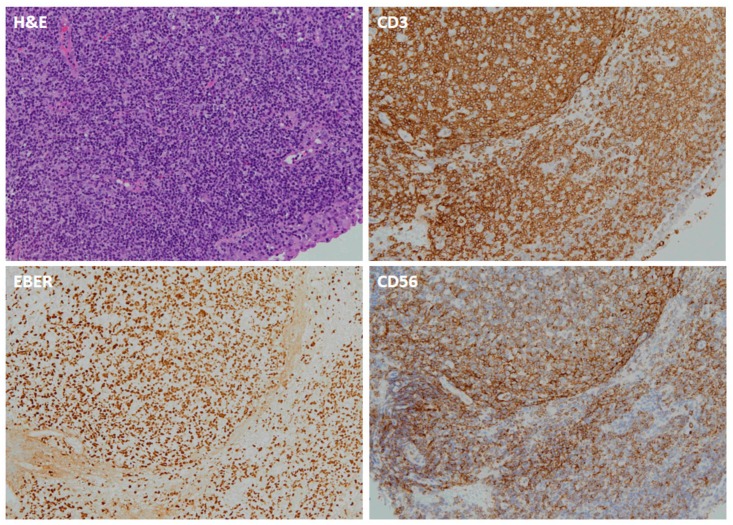
Pathologic images of NP-NKTCL. The H&E-stained slide shows diffuse proliferation of small to medium sized lymphoid cells. The neoplastic lymphoid cells are positive for CD3, CD56, and EBER.

Regarding EBER staining, 13 cases were EBER-positive, including 7 cases of NKTCL, 5 cases of DLBCL, and 1 case of PTLD (Table 1) (Figure 4 and Figure 5). There were three cases for which EBER staining was not possible because of insufficient specimen, including one case of DLBCL and two cases of NKTCL. 

### 3.3. Treatment Modality

Most of the patients (94.3%, *n* = 33) received chemotherapy (CT). Only one patient received radiotherapy (RT) alone; 22.9% of patients (*n* = 8) received both CT and RT. Some patients (22.9%, *n* = 8) also received peripheral blood stem cell transplantation (PBSCT) after primary treatment. 

### 3.4. Survival

Although there was no significant difference of OS between patients receiving CT alone or combined chemoradiotherapy, a higher 10-year OS was observed in patients receiving combined therapy (41.7% vs. 31.2%). Fewer than half of the patients (*n* = 16) were alive at the last follow-up. Two-year, 5-year, and 10-year OS were 67.9%, 50.4%, and 33.6%, respectively. OS was not correlated with sex (*p* = 0.816), B symptoms (*p* = 0.305), age (*p* = 0.414), and Ann Arbor stage (*p* = 0.959). 

### 3.5. Recurrence

There were eight patients (22.9%) who experienced recurrence, including five patients (62.5%) with neck recurrence, two patients with distant recurrence (lung and thigh), and one patient with local (NP) recurrence. 

### 3.6. Subgroup Analysis

**Figure 6:**
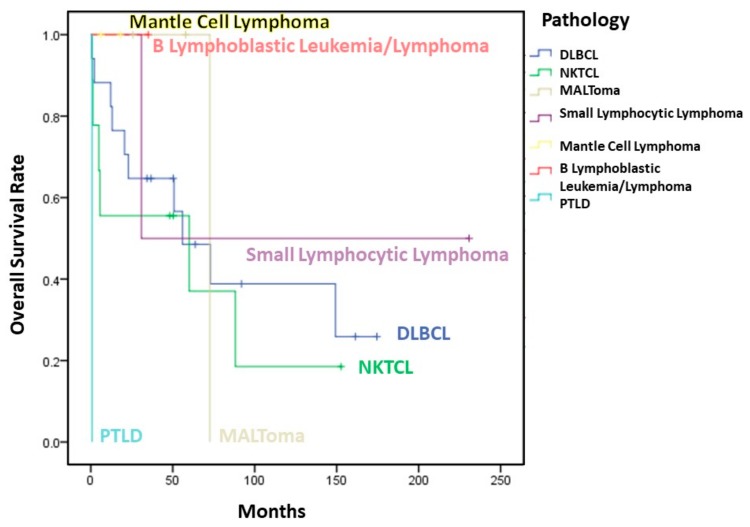
Overall survival of 35 patients with NP lymphoma in our study.

A comparison of the main clinical characteristics of NP DLBCL (NP-DLBCL) and NKTCL (NP-NKTCL) patients is shown in Table 2. The ratio of males to females was 1.125:1 in NP-DLBCL and 2:1 in NP-NKTCL. Patients with NP-DLBCL tended to present with regional lymph node involvement and stage IV disease. Among several clinical characteristics, only duration of symptoms and EBER expression had significant differences between the two groups. The mean duration of symptoms in NP-DLBCL patients was 1.53 months, while the mean duration of symptoms in NP-NKTCL patients was 4.33 months (*p* = 0.009). For cases with EBER ISH data (*n* = 32), all NP-NKTCL revealed EBER positivity (*n* = 7), while 5 out of 16 cases of NP-DLBCL showed EBER positivity. More cases of NP-DLBCL received CT only (75% vs. 55.6%), while more cases of NP-NKTCL received combined CT and RT (44.4% vs. 25%). The mean OS rate of NP-DLBCL patients was 84.4%, which was higher than the mean OS rate of NP-NKTCL patients (57.2%), but this difference was not statistically significant (*p* = 0.413) (Table 2) (Figure 6).

## 4. Discussion

NP lymphoma is a rare extranodal hematologic malignancy with a variety of underlying histopathologic subtypes. Although the incidence of the subtypes of NP lymphoma has been reported to be different in different areas of the world [2,3,4,5,6,7,9,10], NP lymphoma is broadly classified into two types, HL and NHL. NHL accounts for the majority of lymphomas, about 86% of lymphoma cases in the Western population and 90% of lymphoma cases in the Asian population [25,26,27]. Among NHL, B cell lymphoma is the most common lymphoma in western countries, making up more than 90% of NHL cases [26,28], but is less common in Asian countries (64.3%) [27]. B cell lymphoma is also naturally more common among NP lymphomas, with 52.5% of cases in the Asian population and 77.5% of cases in the Western population, respectively [7,9]. In contrast, NKTCL accounts for 47.5% of NP lymphomas in the Asian population, while it accounts for only 6% of NP lymphoma in the Western population [7,9].

The WR is an uncommon site for NHL, with only 10–18% of NHL in Asia occurring in WR in Asia [5,29,30]. In Wu et al.’s study, NP presented as the primary site of tumor location in 26% of WR-DLBCL and in 66% of WR-NKTCL. Regarding NP lymphoma, the most common histological type was reported to be B-cell lymphoma (48.6–87.6% of NP lymphoma) [2,6,9,31]. In our study, the most common NP lymphoma was also DLBCL (48.6%), followed by NKTCL (25.7%), and poorer prognosis was noted for NKTCL compared to DLBCL. Other rare subtypes of NP lymphoma included MALToma (8.6%), small lymphocytic lymphoma (5.7%), mantle cell lymphoma (5.7%), B lymphoblastic leukemia/lymphoma (2.9%), and PTLD (2.9%) (Figure 1). Han et al reported that 2.9% of NP lymphoma cases were HL [9]. However, there were no cases of HL in our study. Compared to the western literature, the incidence of NKTCL in our study was relatively high [9] (Table 3).

In our study, the average age of the patients was 59.6 years, and there was a slight male preponderance, similar to previous studies of NP lymphoma [2,3,4,9]. However, we did not find any correlation between sex or age and survival, which is different from what previously reported [2,9,32]. These conflicting results may be due to different sample sizes and races examined in different studies.

Symptoms and signs of NP lymphoma include neck mass (69–85%), epistaxis (19-33%), hearing loss (8–23%), nasal discharge (15–51%), and nasal obstruction (50–88%) [3,6]. Since all of these symptoms may occur in patients with any NP mass, it is important to differentiate NP lymphomas from other NP tumors. The major difference is that some patients with NP lymphomas may suffer from constitutional symptoms (18–27%), the so-called B symptoms, including weight loss, night sweats, and fevers, which are less common in patients with other NP tumors [3,6,9]. However, some patients may have no symptoms at diagnosis [33]. Naturally, the only method for a definite diagnosis is a biopsy-based pathological analysis. In our study, the common symptoms included nasal obstruction (28.9%), epistaxis (25.7%), neck mass (22.9%), purulent rhinorrhea (17.1%), headache (14.3%), and B symptoms (14.3%). Cervical nodal involvement was relatively limited in our study compared to other studies [6]. Moreover, neck lymph node involvement was only found in cases with NP-DLBCL. B symptoms have been reported to be associated with poor prognosis [34], but this correlation was not confirmed in our study. 

The average duration of the symptoms before the diagnosis of NP lymphoma was not mentioned in related studies. However, there was a study regarding a lymphoma of the nasal cavity and paranasal sinuses, in which the average duration of the symptoms was 5.9 months prior to diagnosis [35]. In the same study, improved survival was found in patients who had symptoms for less than 3 months (70%) compared with those who had symptoms for more than 3 months (25%) [35]. In our study, the average time from initial symptoms to diagnosis ranged from 0.5 to 12 months (mean 2.6 months), and there was a significant difference of symptom duration between NP-DLBCL and NP-NKTCL (1.53 months vs. 4.33 months, *p* = 0.009). Because most symptoms of NP lymphoma are non-specific, patients may be diagnosed late when they are at an advanced stage. Therefore, it is important to be cautious when approaching patients with these symptoms.

In southern Asia, NPC is a relatively common malignancy. Its incidence in southern China is estimated to be approximately 10–50 cases per 100,000 [36], while the incidence is rare in Western countries, around 2.2/100,000 [37]. In Taiwan, the latest cancer registry data released by the government showed that the incidence of NPC is 3.2–9.8 per 100,000, while the incidence of NHL is around 9.1–11.9 per 100,000 [38]. Therefore, it is important to differentiate NP lymphomas from NPC in patients presenting with NP tumors in endemic areas with a high incidence of NPC, such as Taiwan. 

Treatments for lymphoma include systemic CT, RT, PBSCT, targeted therapy (e.g., anti-CD20), and combined therapy [39]. B-Cell NHL has been found to be more sensitive to anthracycline-based CT than NKTCL [40]. Wu et al. also found that WR-DLBCL was more responsive to chemotherapy than WR-NKTCL [7]. The suitable treatment modality usually depends on the subtype of the lymphoma. Miller et al. reported that CT followed by involved-field RT was superior to CT alone for the treatment of localized intermediate- and high-grade NHL [41]. Aviles et al. focused on WR-NHL and revealed that OS was better for patients in the combined-therapy arm (90%) than for those in the CT arm (58%) and RT arm (56%) [42]. Han et al. has proposed that RT is a positive prognostic indicator for NP lymphomas, with the exception of NKTCL [9].

Because this is a retrospective study, there were heterogeneous treatment modalities for the NP lymphoma patients. In our study, more than 90% (*n* = 33) of patients received CT, while only one patient diagnosed with MALToma received RT alone, and this patient had a 25.7-month disease-free survival time. Among patients who received CT (*n* = 33), 24.2% of them (*n* = 8) also received RT concomitantly or sequentially. More cases of NP-DLBCL received CT alone, while more cases of NP-NKTCL received combined CT and RT. The five-year OS was 54.5% in the CT group and 41.7% in the combined chemoradiotherapy group, but there was no significant difference. However, a higher 10-year OS was observed in patients who received combined therapy. In subgroup analysis, the OS of DLBCL patients receiving CT and combined chemoradiotherapy were 90.0 months and 87.0 months, respectively (*p* = 0.920). In the subgroup of NKTCL, the OS of CT-treated and combined chemoradiotherapy-treated patients was 36.7 months and 81.2 months, respectively (*p* = 0.260). Interestingly, studies regarding the Western population showed no evidence of survival benefit from RT for DLBCL patients, but a trend of improved survival after combined chemoradiotherapy was found for our NKTCL patients [43]. 

Regarding OS of NP lymphomas, Han et al. [9] reported a five-year OS of 57%, while Allam et al. [6] reported a two-year OS rate of 75%, and Laskar et al. reported a five-year OS of 57.9% [3]. In our study, the 2-year, 5-year, and 10-year OS rates were 67.9%, 50.4%, and 33.6%, respectively, which is similar to what reported in other studies. The mean OS of NP-DLBCL and NP-NKTCL patients were 84.4% and 57.2%, respectively, but without statistically significant difference. Similar to Zou et al.’s study, worse survival was found in NP-NKTCL patients compared to patients with early-stage NP B-cell lymphoma (five-year OS: 35.5% vs. 69.5%, *p* = 0.003) [5]. Yang et al. classified early-stage NKTCL patients into low- and high-risk groups based on age, Eastern Cooperative Oncology Group (ECOG) status, stage II disease, LDH levels, and primary tumor invasion (PTI) [44]. The study found that primary RT improved survival for early-stage NKTCL patients [44]. RT followed by CT improved survival in the high-risk group. In the low-risk group, the five-year OS rate was 88.8% for patients receiving RT alone and 86.3–86.9% for patients receiving combined CT and RT, while it was 72.2% for patients receiving CT after RT and 58.3–59.6% for patients receiving RT after CT or RT alone in the high-risk group [45]. Lee et al. reported that in Ann Arbor stage I–II sinonasal DLBCL patients, there was no difference in survival between patients receiving CT alone and those receiving combined CT and RT [43]. In our study, for the NP-NKTCL patients, superior survival was observed for patients receiving combined CT and RT compared to the patients receiving CT alone (81.7% vs. 36.9%). However, similar OS was observed for patients with NP-DLBCL, no matter whether the patient received combine CT and RT or CT alone. This implies that NP-NKTCL might be more sensitive to RT than NP-DLBCL. 

Recurrence rate and sites of recurrence are highly variable in the literature [2,6]. In our study, eight cases (22.9%) had recurrence, including two cases (20%) of DLBCL and one case (11.1%) of NKTCL. According to Wu et al., 50% of patients with WR-DLBCL and 64% of patients with WR-NKTCL had relapse at extranodal sites, with WR-DLBCL having significantly more extensive lymph node involvement than WR-NKTCL [7]. Lee et al. proposed that the relapse rate for DLBCL was 13.8%, with local recurrence being the most common recurrence type [43]. Interestingly, for the eight recurrent cases in our study, the most common recurrence site was the neck (62.5%), and only one patient had local recurrence (12.5%).

EBER positivity for both NPC and NKTCL is very high. However, the frequency of EBER positivity for DLBCL in different geographic regions varies, being higher in Asian countries (8% ± 9%) than in some Western countries (1% ± 3%) [10,18,19,20,21,22]. Koh et al. reported that, in classic HL patients, EBER-positive patients had lower OS than EBER-negative patients [23]. A meta-analysis including 13 studies found that EBER positivity was correlated with adverse clinicopathologic features, worse clinical course, and poor outcome [10]. Plasma EBV DNA, derived from the apoptosis of lymphoma cells, is reported to be a surrogate biomarker of lymphoma load and may provide prognostic information [46,47]. EBV-associated viral proteins were found to play a crucial role in oncogenesis, including in the regulation of proliferation, immune escape, metastasis, and epigenetic modifications [10,14,15,16,17]. Constitutive expression of latent membrane protein 1 (LMP1) was found to lead to activation of NF-kB and JAK/STAT signaling, which protects against apoptosis and activates autocrine/paracrine signals. This further promotes tumor growth and immune evasion [17]. Additionally, previous studies proposed that EBV may induce angiogenesis in the lymphoid tissue when LMP-1 activates angiogenetic factors [23]. Our study is the second study to focus on EBER expression in NP lymphoma. For cases (*n* = 32) with EBER ISH staining results, 13 cases showed EBER positivity, including cases of NKTCL (*n* = 7, 100% of NKTCL cases with EBER ISH staining) (Figure 5), DLBCL (*n* = 5, 31.3% of DLBCL cases with EBER ISH staining) (Figure 4), and PTLD (*n* = 1). The frequency of EBER positivity for DLBCL was higher than in other studies. The five-year OS of EBER (+) and EBER (−) cases was 55.6% and 58.5%, respectively. In the DLBCL subgroup, five-year OS for EBER (+) and EBER (−) cases was 50% and 85.7%, respectively. Although there was no significant difference, EBER-positive DLBCL patients had poorer prognosis compared to EBER-negative DLBCL patients. The relationship of EBV infection with prognosis in NP lymphoma needs further investigation in the future.

EBER ISH has been considered as the gold standard for evaluating EBER positivity for many years. It allows a direct visualization of the neoplastic cells. However, it is a semi-quantitative analysis, and subtle differences in technical quality cannot be eliminated [10]. More sensitive methods, such as PCR for EBV viral load measurement, EBV-encoded miRNAs detection, and RNAscope assay, may help to confirm whether there is EBV infection in EBER-negative samples [48,49]. However, the NP tissue for PCR analysis should contain only tumor tissue, to avoid biased results.

Gene expression profıling can classify DLBCL into two clinically distinct subgroups, i.e., germinal center B-cell (GCB) and non-GCB, which includes activated B-cell (ABC) [50,51]. The GCB subtype is associated with better outcomes, while the ABC subtype is associated with worse outcomes [52]. Hans et al. divided DLBCL into GCB and non-GCB subgroups by using immunostaining for CD10, bcl-6, and MUM1 and found an outcome similar to that predicted by cDNA microarray analysis [53]. Since our study is a retrospective study, we did not perform a cDNA microarray analysis to divide our DLBCL cases into GCB or ABC. However, several cases were studied by IHC for CD10, bcl-6, and MUM1 expression. Among these, there were one GCB and seven non-GCB lymphomas according to Hans criteria (Supplementary Table S2). This may explain the relatively poor outcomes of our DLBCL cases. Besides, a high proportion of stage IV patients (*n* = 5, 29.4%) were noted in our DLBCL patients. None of our DLBCL patients relapsed with CNS disease. 

NPC is also highly related to EBV, and more than 90% of NPC are EBER-positive. Therefore, it is important to differentiate NP lymphomas from NPC, especially in areas with a high incidence of NPC. NPC usually originates from the posterolateral nasopharynx at the fossa of Rosenmüller, while NP lymphoma usually presents as diffuse swelling of NP under nasal endoscopic examination (Figure 3). The surface of NP lymphomas may be inflamed, with a central necrotic area. According to Liu’s research, NP lymphoma can be distinguished from NPC by MR imaging, for NPC tumor lesions are more likely to be asymmetrical and invade more deeply into tissues, while NP lymphomas are usually symmetric and tend to spread laterally [36]. Otolaryngologists should be wary about making a diagnosis of NP lymphoma, especially in areas endemic with NPC.

There are only few studies reporting the clinical characteristics and prognosis of NP lymphomas, and most of them studied T-cell and B-cell lymphomas as a single group [2,3,4,6,8,9]. Very few studies have specifically addressed the clinical disparities of NP-DLBCL and NP-NKTCL. Our study demonstrated the differences between the two types and also investigated EBER expression in NP lymphoma, especially in an area with high NPC occurrence. However, one limitation of this study is that there was no certain way to determine whether stage IV disease originated from the nasopharynx primarily. Larger-scale studies are needed in the future to better understand the development of this rare disease.

## 5. Conclusions

NP lymphoma is a rare malignancy developing in the head and neck region and encompasses a variety of histological subtypes. NP-DLBCL and NP-NKTCL are the two most common subtypes, and there are several differences between them regarding clinical features and prognosis. In endemic areas with a high frequency of NPC occurrence, NP lymphoma should always be considered in the differential diagnosis when approaching a patient with a nasopharyngeal mass. 

## Figures and Tables

**Table 1:** Demographic, clinical, and histological information of patients with NP lymphoma.

Case Number (*n* = 35)		
**Age**		17–88 (mean 59.57 ± 18.84)
**Sex**		M/F = 20/15
**Symptoms**		
	Nasal obstruction	10 (28.6%)
	Epistaxis	9 (25.7%)
	Neck mass	8 (22.9%)
	Purulent Rhinorrhea	6 (17.1%)
	Headache	5 (14.3%)
	B symptoms	5 (14.3%)
**Symptom Duration**		0.5–12 (mean 2.61 ± 2.97)
**Location of Disease**		
	NP only	22 (62.9%)
	NP + neck/distant LN ^*^	10 (28.6%)
	NP + adjacent organ ^#^	3 (8.6%)
**Comorbidity**		
	HTN	10 (28.6%)
	DM	6 (17.1%)
	CAD	4 (11.4%)
**Lab**		
	WBC	2400–167800 (mean 12992)
	LDH	106–1702 (mean 337.7)
**EBER**		
	Positive	13 (37.1%)
	Negative	19 (54.3%)
	NA	3 (8.6%)
**Stage (Ann Arbor)**		
	I	12 (34.3%)
	II	15 (42.9%)
	IV	8 (22.9%)
**Treatment**		
	CT	20 (57.1%)
	RT	1 (2.9%)
	CT+RT	6 (17.1%)
	CT+PBSCT	5 (14.3%)
	CT+RT+PBSCT	2 (5.7%)
	No treatment	1 (2.9%)
**Recurrence**		8 (22.9%)
**Dead**		19 (54.3%)

# sinonasal cavity, oropharynx, hypopharynx; *gastrointestinal (GI) tract, inguinal lymph node (LN), neck LN. Abbreviation: CT: chemotherapy; RT: radiotherapy; PBSCT: peripheral blood stem cell transplantation; EBER: Epstein–Barr virus-encoded small RNAs; NA: not available; HTN: hypertension; DM: Diabetes mellitus; CAD: Coronary artery disease; WBC: white blood cell count; LDH: lactic dehydrogenase.

**Table 2:** Demographic and histopathological characteristics of NP-DLBCL and NP-NKTCL patients.

	DLBCL(n = 17)	NKTCL(n = 9)	
**Characteristic**	No (%)	No (%)	*P* value
**Sex**			0.683
male	9 (52.90%)	6 (66.70%)	
female	8 (47.10%)	3 (33.30%)	
**Age**	61.59+/-22.49 (17-88)	59.44+/-17.27 (38-81)	0.806
**Duration**(months)	1.53+/-0.86 (0.5-4)	4.33+/-3.94 (1-12)	**0.009**
**Stage**			0.324
I	4 (23.50%)	5 (55.60%)	
II	8 (47.10%)	3 (33.30%)	
IV	5 (29.40%)	1 (11.10%)	
**Location of Disease**			0.822
NP only	11 (64.70%)	7 (77.80%)	
NP+ neck/distant LN *	4 (23.50%)	1 (11.10%)	
NP+ adjacent organ #	2 (11.80%)	1 (11.10%)	
**EBER ^†^**			**0.005**
Positive	5 (31.25%)	7 (100%)**^†^**	
Negative	11 (68.75%)	0 (0%)	
**B symptoms**	1 (5.90%)	2 (22.2%)	0.268
**Cervical Node Involvement**	4 (23.50%)	0 (0%)	0.263
**Treatment**			0.394
CT only	12 (75%)	5 (55.60%)	
CT+RT	4 (25%)	4 (44.40%)	
Recurrence *	4 (23.53%)	1 (11.10%)	0.628
**Dead**	10 (58.82%)	6 (66.70%)	1
**Overall survival**	25.90%	16.10%	0.515
3-year	64.70%	55.60%	0.505
5-year	48.50%	55.60%	0.803
10-year	38.80%	18.50%	0.417

# sinonasal cavity, oropharynx, hypopharynx, * GI tract, inguinal LN, neck LN. **^†^** There were 3 missing data, 1 of DLBCL and 2 of NKTCL.

**Table 3:** Summary of studies of NP lymphoma.

Year	Author	Country	No.	Age	Sex (M/F)	Tumor Pathology			EBER (+)	Treatment			Survival (OS)
						DLBCL	NKTCL	Other		CT	RT	CT + RT	
1999	Lei [2]	China	19	51	15/4	11(57.9%)	5(26.3%)	3(15.8%)	NA	6(32%)	2(11%)	11(58%)	5-Y OS:82% 5-Y DFS:76%
2006	Laskar [3]	India	113	40	79/34	94(83.2%)	0 T-cell	19(16.8%)	NA	25(22%)	0(0)	86(76%)	5-Y OS:57.9% 5-Y DFS:55.8% 2 expired before treatment
2006	Zou [5]	China	80	NA	NA	48(60%) B-cell	32(40%)	0	NA	31(38.8%)	7(8.8%)	42(84%)	5-Y OS B-cell origin: 69.5% NKTCL: 35.5% 5-Yprogression-free survival: B-cell origin: 53.3% NKTCL: 28.9%
2006	Mitarnun [4]	Thailand	42	57.2	24/18	35(83.3%)	3(7.1%)	4(9.5%)	9/42 (21.4%)	NA	NA	NA	NA
2009	Allam [6]	Morroco	26	52.7	NA	13(50%)	4(15.4%) T cell	9(34.6%)	NA	27%	0(0)	73%	1-Y OS:87% 1-Y DFS: 71% DLBCL:2-Y OS:75%
2014	Wu [7]	China	61	49 all WR	NA	32(52.5%)	29(47.5%)	0	NA	NA	NA	NA	WR-NKTCL 5-Y OS:68% WR-DLBCL 5-Y OS: 74%
2017	Han [9]	USA	1119	59.3	658/461	867(77.5%) B-cell	67(6.0%)	185(16.5%)	NA	NA	452(41.5%)	NA	2-Y OS:70% 5-Y OS:57% 10-Y OS:45% 2-Y DSS:77% 5-Y DSS:68% 10-Y DSS:62%
2019	Hsueh	Taiwan	35	59.6	20/15	17(48.6%)	9(25.7%)	9(25.7%)	13/32 (37.1%)	25(71.4%)	1(2.9%)	8(22.9%)	DLBCL: 3-Y OS:64.7% 5-Y OS:48.5% 10-Y OS:38.8% NKTCL: 3-Y OS:55.6% 5-Y OS:55.6% 10-Y OS:18.5%

Abbreviations: WR: Waldeyer’ ring; DLBCL: diffuse large B cell lymphoma; NKTCL: NK/T cell lymphoma; NP: nasopharynx; CT: chemotherapy; RT: radiotherapy; OS: overall survival; DSS: disease specific survival; DFS: disease free survival; EBER: Epstein–Barr virus-encoded small RNAs.

## Supplementary Materials

The following are available online at https://www.mdpi.com/2077-0383/8/10/1604/s1, Table S1: Characteristics of stage IV NP lymphoma patients; Table S2: IHC and subtypes of NP DLBCL patients.
