# Supplementary material for: Nasopharyngeal Lymphoma: A 22-Year Review of 35 Cases
Source: J Clin Med. 2019 Oct 3;8(10):1604. doi: 10.3390/jcm8101604 (PMC6833098; doi:10.3390/jcm8101604)
Supplement: Supplementary file 1 [file jcm-08-01604-s001.pdf]

Supplementary Table S1. Characteristics of Stage IV NP lymphoma patients

No.	Age/Sex	Pathology	EBER	Other involved sites	Survival (Months)
1	62/M	Mantle cell lymphoma	-	*GI tract, *bone marrow, mediastinum, retroperitoneum, spleen, mesentery,	6.2
2	50/F	DLBCL	+	Tonsil, neck LN, , mediastinum, lung, stomach, retroperitoneum, pelvic and *inguinal regions	34.3
3	56/F	PTLD	+	Left thigh, lung, abdomen	0.8
4	88/F	DLBCL	+	Lung, pelvic, *inguinal region, left deltoid	91.9
5	50/M	DLBCL	NA	Inguinal region, submandibular, paraaortic, retroperitoneum, left external iliac chain	12.1
6	17/M	DLBCL	-	Axillary, mediastinum, retroperitoneal, inguinal LN, bone marrow	161.4
7	72/F	DLBCL	+	NA	50.8
8	81/M	NKTCL	NA	Lung, abdomen	0.9

Abbreviations: DLBCL: diffuse large B cell lymphoma; EBER: Epstein–Barr virus-encoded small RNAs; LN: lymph nodes; NA: not available; NP: nasopharynx; NKTCL: NK/T cell lymphoma; PTLD: post-transplant lymphoproliferative disorder

* Biopsy-proven pathology

Supplementary Table S2. IHC and subtypes of NP DLBCL patients

No.	CD10	BCL-6	MUM1	Subtype	Survival (Months)	CNS involvement	EBER
1	-	+	+	Non-GCB	34.3	-	+
2	-	+	+	Non-GCB	20.6	-	_
3	-	+	+	Non-GCB	36.8	-	_
4	-	+	+	Non-GCB	50.3	-	+
5	-	+	+	Non-GCB	63.7	-	_
6	-	+	NA	NA	91.9	-	+
7	-	+	NA	NA	73.1	-	_
8	-	-	NA	Non-GCB	0.7	-	+
9	-	+	NA	NA	2.1	-	_
10	+	+	NA	GCB	12.1	-	NA
11	-	+	+	Non-GCB	23.0	-	_
12	NA	NA	NA	NA	161.4	-	_
13	-	+	NA	NA	56.0	-	_
14	NA	NA	NA	NA	50.8	-	+
15	NA	NA	NA	NA	174.5	-	_
16	-	+	NA	NA	13.0	-	_
17	NA	NA	NA	NA	149.3	-	_

Abbreviations: DLBCL: diffuse large B cell lymphoma; GCB: germinal center B-cell; EBER: Epstein–Barr virus-encoded small RNAs; NA: not available
